# Ethical challenges in global research on health system responses to violence against women: a qualitative study of policy and professional perspectives

**DOI:** 10.1186/s12910-024-01034-y

**Published:** 2024-03-19

**Authors:** Natalia V. Lewis, Beatriz Kalichman, Yuri Nishijima Azeredo, Loraine J. Bacchus, Ana Flavia d’Oliveira

**Affiliations:** 1 Centre for Academic Primary Care, Population Health Sciences, Bristol Medical School, University of Bristol, Bristol, UK; 2 Department of Preventive Medicine, Medical School, University of São Paulo, São Paulo, Brazil; 3 Department of Global Health and Development, London School of Hygiene and Tropical Medicine, London, UK

**Keywords:** Violence against women, Health research, International collaboration, Global health, Ethics, Research ethics, Ethical issues, Policy, Qualitative study

## Abstract

**Background:**

Studying global health problems requires international multidisciplinary teams. Such multidisciplinarity and multiculturalism create challenges in adhering to a set of ethical principles across different country contexts. Our group on health system responses to violence against women (VAW) included two universities in a European high-income country (HIC) and four universities in low-and middle-income countries (LMICs). This study aimed to investigate professional and policy perspectives on the types, causes of, and solutions to ethical challenges specific to the ethics approval stage of the global research projects on health system responses to VAW.

**Methods:**

We used the Network of Ethical Relationships model, framework method, and READ approach to analyse qualitative semi-structured interviews (*n* = 18) and policy documents (*n* = 27). In March-July 2021, we recruited a purposive sample of researchers and members of Research Ethics Committees (RECs) from the five partner countries. Interviewees signposted policies and guidelines on research ethics, including VAW.

**Results:**

We developed three themes with eight subthemes summarising ethical challenges across three contextual factors. The global nature of the group contributed towards power and resource imbalance between HIC and LMICs and differing RECs’ rules. Location of the primary studies within health services highlighted differing rules between university RECs and health authorities. There were diverse conceptualisations of VAW and vulnerability of research participants between countries and limited methodological and topic expertise in some LMIC RECs. These factors threatened the timely delivery of studies and had a negative impact on researchers and their relationships with RECs and HIC funders. Most researchers felt frustrated and demotivated by the bureaucratised, uncoordinated, and lengthy approval process. Participants suggested redistributing power and resources between HICs and LMICs, involving LMIC representatives in developing funding agendas, better coordination between RECs and health authorities and capacity strengthening on ethics in VAW research.

**Conclusions:**

The process of ethics approval for global research on health system responses to VAW should be more coordinated across partners, with equal power distribution between HICs and LMICs, researchers and RECs. While some of these objectives can be achieved through education for RECs and researchers, the power imbalance and differing rules should be addressed at the institutional, national, and international levels. Three of the authors were also research participants, which had potential to introduce bias into the findings. However, rigorous reflexivity practices mitigated against this. This insider perspective was also a strength, as it allowed us to access and contribute to more nuanced understandings to enhance the credibility of the findings. It also helped to mitigate against unequal power dynamics.

**Supplementary Information:**

The online version contains supplementary material available at 10.1186/s12910-024-01034-y.

## Introduction

Violence against women (VAW) is a global public health and clinical problem leading to increased mortality and morbidity among women and their children [1]. Globally, 27% of ever-partnered women aged 15–49 years have experienced physical and/or sexual intimate partner violence in their lifetime, with 13% experiencing it in the past year. Low-income countries reported higher prevalence compared with high-income countries [2]. Health systems have a crucial role in a multisector response to VAW through identifying and supporting people who have experienced violence [3]. Prior research identified considerable system-, organisation-, and individual- level barriers to health system responses to VAW, especially in low-income and middle-income countries (LMICs) [4] and proposed a framework for improving health system readiness to address VAW [5]. In the past decade, governments and other funders in high-income countries (HIC) made substantial investments in global research addressing the Sustainable Development Goals, including elimination of VAW [6].

Studying VAW as a global public health and clinical problem requires collaboration between researchers from different disciplines and countries. Such multidisciplinary and multiculturalism create challenges in adhering to a single set of ethical standards applied across differing country-specific contexts characterised by power and resource inequalities. Research activities happen in the contexts which reflect both global and local cultural and social dynamics, with research ethics regulations varying not only across countries but also across fields of knowledge which means that multidisciplinary multicounty research is bound to face specific challenges. Members of global research groups are embedded within their teams and organisations which have differing resources, structures, cultures and politics. The organisations are influenced by the differing economic, social, and political environments. The provision of funding and research capacity from HICs to LMICs exacerbates existing power imbalances. Which ethical standards hold precedence – those developed by the international community, the HIC funder and grant holding institution, the LMICs where the research is taking place, or all the above?

Studies on VAW fall into the category of sensitive topics because they impose additional emotional burden and threat to physical and social self of participants and researchers. The sensitivity of the VAW research is also determined by the exploration of culturally and politically rooted issues of social control, coercion and domination, interests of powerful people, the ‘sacred’ concepts of family relations and power, and the lived realities of people who have experienced or used violence [7]. The increased sensitivity surrounding the topic of VAW gives rise to additional ethical dilemmas concerning the principles of respect for persons, confidentiality, justice, beneficence, and nonmaleficence [8]. Global groups studying health system response to VAW should resolve these dilemmas while applying international-, funder-, and country-specific ethical requirements to the sociocultural and economic context, VAW services and health systems in LMICs. How can LMIC researchers adhere to all the ethical requirements while protecting their cultural diversity and the safety of their research participants, communities, and researchers?

Previous studies have acknowledged ethical challenges in global research [9] including studies on VAW [8, 10]. Ethical and methodological challenges in global research on VAW are interlinked and both can undermine the quality of the data and findings [11, 12]. Recent theoretical developments equipped researchers with tools for exploring and addressing ethical challenges in global research. Reid et al. [13] created the ‘4Ps’ model: Place, People, Principle and Precedent—for analysing and developing solutions to ethical conflicts in global research. Morrison et al. [14] developed the Network of Ethical Relationships (NER) model in the context of global population health research. NER identified relational challenges within research teams, with Research Ethics Committees (RECs), funders, and participants which were embedded in the complex and conflicting normative framework regarding HIC and LMIC legal rules, societal norms, moral values, and institutional rules. The ethical relationship challenges were explained by differing cultural backgrounds, REC requirements and participant values, conflicting requirements between HIC and LMIC RECs and funding procedures. However, to our knowledge, no studies have addressed ethical challenges in global research on health system response to VAW. This study aimed to investigate professional and policy perspectives on the types, causes of, and solutions to ethical challenges specific to the REC approval stage of a global research programme on health system responses to VAW.

### Study context: the global health research group

**Fig. 1:**
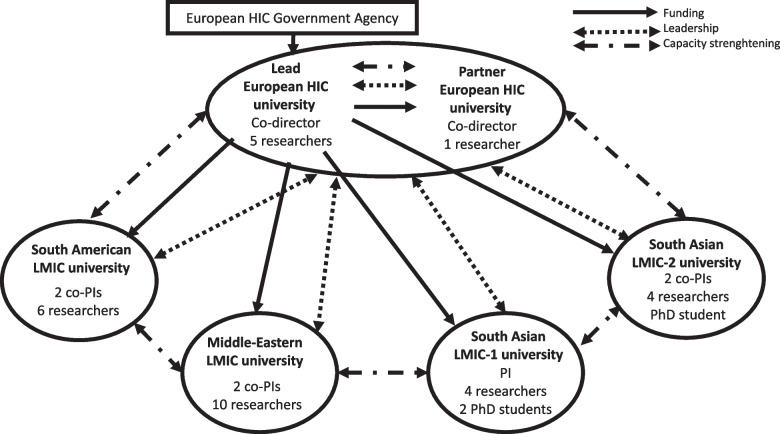
Global research group on health system responses to violence against women in low- and middle-income countries

This paper draws on our experience as a global research group on health system responses to VAW in LMICs. The partnership included two universities in a European HIC and four LMIC universities (one South American, one in the Middle-Eastern region, and two in different South Asian countries). The group, funded by the government agency in the European HIC, aimed to: (i) develop and pilot-test LMIC-specific interventions in sexual and reproductive health services addressing VAW, (ii) strengthen the research capacity of HIC and LMICs universities, (iii) evaluate capacity strengthening activities (Fig. 1).

The countries were diverse amongst themselves, with different health systems and research infrastructures, which shaped all aspects of the research, including ethics approval. At macro level, the HIC funding agency dictated the financial and management structure of the global research group. The funder created conditions for maintaining power at the lead contracting university in the HIC, which held the grant and distributed funds quarterly to the five partner universities. Two group co-directors were also based at the HIC universities. Each LMIC university had a local principal investigator or co-principal investigators with a team of researchers and PhD students responsible for country-specific primary studies. HIC researchers supported the study designs and methodological development, were involved in the capacity strengthening workstream, and led on syntheses of findings from LMIC primary studies.

At meso level, the composition of the group also created conditions for maintaining power at HIC universities because their researchers had more methodological expertise and experience. However, researchers in both HIC universities and two LMIC universities brought together extensive expertise and experience in the VAW topic. We proactively explored and addressed potential power imbalances through meetings and research capacity strengthening activities. As described in a separate publication, we carried out a baseline evaluation and mapping exercise of the research capacities within and across all country teams [15]. The evaluation showed that while the HIC teams included more mid-career and senior researchers with extensive methodological expertise in health system responses to VAW, their knowledge of the health systems and socio-cultural-historical contexts in the partner LMICs was limited. In contrast, LMICs teams had a greater proportion of early career researchers and less expertise in some research methods. However, they were well embedded within the local communities and health care systems where the primary research was conducted. They also possessed a high degree of local knowledge regarding power dynamics between different stakeholders, processes for engaging with them, and political and cultural sensitivity. It is important to acknowledge that the power imbalance was partly influenced by the nature of the work being done by the different teams. The LMICs partners were primarily responsible for fieldwork, a role typically assigned to early career researchers, whereas the HIC partners focussed more on supporting instrument development, data analysis, and capacity strengthening workpackage requiring researchers with greater experience.

To reflect on the power imbalances between the teams, we organised a participatory workshop with researchers from all country teams [15]. We agreed on shared values, identified barriers, and planned capacity strengthening activities. The shared values included: mutual learning, respect, fair opportunity, clear boundaries, honesty, and transparency. LMIC researchers identified barriers related to limited methodological expertise, access to training courses, information technologies and English-language skills for academic writing which we targeted through the capacity strengthening activities. We mapped areas of methodological expertise within countries and identified opportunities for mentoring and mutual learning across partners. For example, the South American team which was involved in developing ethical principles for the WHO Multi-Country Study on Women’s Health and Domestic Violence Against Women [16] delivered ethics training to the whole group. Researchers from the Middle-Eastern and European teams co-delivered a workshop on measurement and routinely collected data. Researchers from the South American and South Asian teams co-led a workshop on how we use feminist principles and theory in our research on VAW. Mutual learning took place through joint development of protocols and research tools for primary studies, early career researchers virtual peer support and education group, virtual monthly team meetings, and annual face-to-face and hybrid workshops in partner countries.

During one of the group meetings, an LMIC researcher raised concerns about the group adopting terminology used by HIC policy makers and funders which perpetuated the existing power imbalance. They argued that term “capacity building” implied a lack of research capacity in LMICs which they found disempowering. This conflicted with the shared values of respect and mutual learning within our group, as well as the extensive experience in VAW research present in the South American and South Asian partner universities. As a result, we revised our terminology and replaced the term ‘capacity building’ with ‘capacity strengthening’.

From the start, the group made efforts to carry out equitable work despite inequitable conditions, whilst navigating variations in institutional research ethics review requirements, and adhering to diverse regulations imposed by academic and health system institutions. To conduct primary studies, we had to obtain ethics approvals from two HIC and four LMIC universities, as well as additional approvals from the health authorities in all LMICs. This process highlighted power dynamics between the different countries and institutions involved. Tensions arose because of the disparate policies and practices. We encountered challenges that were not previously reported in literature. The two HIC university RECs had conflicting requirements regarding the sequence of ethics approvals among group partners. The REC at the lead HIC university encouraged a local ethics review where possible because the local REC would possess the most relevant expertise to assess the ethics application for research undertaken in the country concerned. This approach aimed to prevent contradictory responses from two separate REC decisions. In contrast, the second HIC university insisted that their REC would require reviewing all studies involving their staff, irrespective of the country involved and whether a local review was already being conducted. They required the local ethics approvals to be provided for their final decision. The conflicting requirements had repercussions on the timely execution of the primary studies in LMICs. The lead HIC REC advised that if the LMIC teams have undergone a local research ethics review and received a favourable ethical opinion, they could commence the research activities specified in their ethics applications and favourable opinions. In contrast, the other HIC REC insisted that the project should not commence until full ethical approval had been obtained from their university, alongside local ethical approval.

Another challenge at the meso level arose from the differing policies and practices for research data management. The HIC funder and two HIC university RECs requested detailed data management plans compliant with the European Union General Data Protection Regulation. However, partners in LMIC were unable to fully comply with the same standards due to different legal requirements in their respective countries and varying policies and processes for research data management within their universities. As a temporary solution, the lead HIC university granted all LMIC principal investigators/co-investigators and their researchers an honorary status enabling them to access secure departmental file storage. The partners signed Data Sharing Agreement and Data Repository Agreement for using Research Data Repository at the lead HIC university for storing and sharing research data which underpinned outputs from the primary studies.

## Methods

### Study design

The international team of researchers with backgrounds in psychology (NVL, YNA), policy (BK), medicine (AFDO, NVL), and social science (LJB) conducted a qualitative study comprising of semi-structured interviews and a document review of ethics policies and guidelines. Our positionality in the critical realism ontology [17] and feminist epistemologies and methodologies [18] influenced the choice of a qualitative research design to explore the contextual factors and processes shaping researchers’ and REC members’ experiences during the ethics approval phase of global research projects on health system responses to VAW. Our approach was also informed by discourses on decolonising global health research [19] and epistemic injustice in academic global health [20]. We recognised the existence of international and institutional hierarchies, that (post)colonial legacies shape the field of global research on VAW and that systemic changes are needed to shift the hierarchies of power [19]. We believed that the experiences and views of the HIC and LMIC researchers and participants were equally credible. We acknowledged that researchers and participants would impact on each other, and that the researchers’ backgrounds would influence data production and analysis. We challenged epistemic injustice through fostering co-creation of knowledge by researchers and study participants with similar experiences. The authors who conducted interviews (NVL, BK) were members of the same global health group; three authors (NVL, LJB, AFDO) were also interviewed as research participants. These authors were not involved in the analysis of their transcripts.

We followed the READ (ready your material, extract data, analyse data, distil your findings) approach for document review [21] and the framework method [22] for data analysis. While interviews explored individual experiences of ethics approval for global research in health system responses to VAW, review of policies and guidelines allowed to contextualise these experiences. Concepts within the NER model were used as sensitising devices which informed the analysis [14].

### Data collection

We conducted online semi-structured qualitative interviews in March-July 2021. Data set size for interviews was informed by the model and concept of information power [23]. We assumed that our study would need less participants because of the narrow aim, high specificity of participants for the study aim, established NER model, strong interview dialog, and cross-case analysis.

We used purposive sampling strategy to recruit researchers and REC members with rich and diverse experience of ethics approvals for global research, representing five partner countries in our global health research group. The study was advertised via an email sent to the group mailing list and snowballed via professional networks. Interested individuals emailed study researchers who confirmed eligibility, provided further information, and arranged interviews on Zoom/Teams. Interviews were conducted in the language of choice of the interviewees. Participants provided verbal informed consent. The topic guides explored experiences of applying ethics policies and guidelines in practice, following REC processes, obtaining ethics approvals, challenges faced, and proposed causes and solutions (Additional file 1). Interviews were audio recorded, professionally transcribed, checked, and anonymised.

We identified policies and guidelines on research ethics through interview participants, electronic searches, and reference checking. Two researchers (NVL, BK) searched websites of the HIC and LMIC universities and RECs involved in the research, as well as of HIC funders and think tanks using terms “violence” “women”, “ethic*”, “guideline”. We retrieved, screened, and selected documents meeting our inclusion criteria: international, national, and institutional policies and guidelines from the five partner countries that discussed global research and/or research on VAW.

### Analysis

We started data analysis while conducting interviews to refine topic guides for further interviews and to identify additional documents. For the document review, we customised an Excel proforma [21] to extract data on title, author, year, source, target audience, key messages, data relevant to global research and studies on VAW. During data extraction, we made notes about how each document addressed ethical issues in global research and/or research on VAW. Interview transcripts and documents were imported into NVIVO 12 for data management and coding. The analysis was conducted using a combination of inductive and deductive approaches. Researchers (NVL, BK, AFDO) worked on their subsets of transcripts and documents in English and local language. These researchers read and re-read two interview transcripts and independently manually coded text relevant to the research questions. The researchers compared initial codes and developed a ‘working analytical framework’ which they then applied to their subsets of transcripts and documents in NVIVO [22]. The framework was refined through four cycles of revisions during coding process. We then grouped our codes into candidate themes, mapped them on the constructs of the NER model in an Excel framework matrix in English, and developed final analytical themes. Researchers (NVL, BK) wrote descriptive accounts of the analytical themes with illustrative quotes. The study team met regularly to discuss the codes, themes, matrix, and descriptive accounts paying attention to the similarities and differences within and between interviews and documents, countries, and institutions.

## Results

We conducted 18 interviews with researchers (*n* = 11) and REC members (*n* = 7) representing all five partner countries and a wide range of professional experience (2–25 years) (Table 1).

**Table 1:** Socio-demographic characteristics of interview participants

Interviewee	Country	Role	Years in role
1	European HIC	University researcher	9
2	European HIC	University researcher	21
3	European HIC	University REC member	10
4	European HIC	University REC member	25
5	South American LMIC	University researcher	23
6	South American LMIC	Municipal REC member	7
7	South American LMIC	University REC member and Hospital REC member	10
8	South American LMIC	University researcher	7
9	South American LMIC	Municipal Health Department REC member	9
10	Middle-Eastern LMIC	University researcher	14
11	Middle-Eastern LMIC	University REC member	10
12	Middle-Eastern LMIC	University REC member	34
13	South Asian LMIC-1	University researcher	2
14	South Asian LMIC-1	University researcher	2
15	South Asian LMIC-1	University researcher	10
16	South Asian LMIC-1	University REC member	9
17	South Asian LMIC-2	University researcher	2
18	South Asian LMIC-2	University researcher	3

*HIC*, high income country, *LMIC* low-and middle-income country, *REC* research ethics committee

Interviews in English (*n* = 15) and local language (*n* = 3) lasted between 27 and 80 min (mean 46 min). Despite support from local researchers, we could not recruit REC members from one South-Asian LMIC. When approached, REC members declined participation explaining that the study was not supported by their institutional and national REC and that it is difficult to speak about issues and challenges which may be against their government. In addition, they felt that this study should have been conducted in collaboration with their RECs and some of the members as co-authors (email correspondence). In contrast, REC at the lead HIC university, transferred our ethics application to a different faculty to prevent conflict of interest.

We analysed 27 documents (4 international, 17 national, 6 institutional), that were categorised into educational material, guidelines, legal documents, policy documents, reports, standard operational procedures, and statements (Table 2).

**Table 2:** Documents included in the analysis

Type of document	Title	Year	Country	Level
**Educational**	Research ethics committees: basic concepts for capacity-building	2009	International	International
	Health Research Training Manual	2015	LMIC-4	National
	Research ethics	2019	LMIC-2	Institutional
**Guideline**	Putting Women First: Ethical and Safety Recommendations for Research on Domestic Violence Against Women	2001	International	International
	International ethical guidelines for health-related research involving humans	2016	International	International
	Guidelines for Institutional Review Committees (IRCs) for Health Research in [LMIC-4]	2016	LMIC-4	National
	National Ethical Guidelines for Health Research in [LMIC-4]	2019	LMIC-4	National
	[HIC Funder] Ethics Guide. Research involving human participants in developing societies	2004	HIC	National
	[HIC Funder] guidelines for management of global health trials. Involving clinical and public health interventions	2019	HIC	National
	[HIC Funder]. Guidance for applicants. Ethics and approvals. Human participants in research	2021	HIC	National
	Pilot REC guidelines	2012	LMIC-2	Institutional
**Legal document**	Law Nº 11.340, 7 of August of 2006	2006	LMIC-1	National
	Resolution nº466, December 12, 1012	2012	LMIC-1	National
	Operational norm 001/2013	2013	LMIC-1	National
	Resolution 510, 4th of April of 2016	2016	LMIC-1	National
**Policy**	National Health Research Policy of [LMIC-4]	2011	LMIC-4	National
	[Research Funder] global health units call 2021. Regulatory approvals and compliance	2021	HIC	National
	Research involving human participants policy	2021	HIC	National
	Ethics Review Committee	Not reported	LMIC-3	Institutional
	[HIC University 1] Ethics of Research Policy and Procedure	2019	HIC	Institutional
	Research Governance and Integrity Policy	2019	HIC	Institutional
**Report**	The ethics of research related to healthcare in developing countries	2002	HIC	National
	The ethics of research related to healthcare in developing countries—a follow-up Discussion Paper	2005	HIC	National
	Building partnerships of equals. The role of funders in equitable and effective international development collaborations	2017	HIC	National
	Research in global health emergencies: ethical issues	2020	HIC	National
**Standard operating procedures**	Standard Operating Procedures. Ethics Review Committee. Faculty of Medicine. [LMIC-3] University	2014	LMIC-3	Institutional
**Statement**	Declaration of Helsinki	2013 update	International	International

*HIC* high income country, *LMIC* low- and middle-income country, *REC* Research Ethics Committee

The reports by HIC funders produced in partnership with researchers from different countries included analysis of global inequalities and consent. The HIC was the only country that wrote documents detailing how to operate as an international research funder, although LMIC had documents detailing international partnerships. Two LMICs had national ethics regulations while all other partners had institutional documents.

Interview participants signposted the same high-level policies and guidelines on ethics in global research: the Helsinki declaration [24], the Council for International Organizations of Medical Sciences’ (CIOMS) International Ethical Guidelines for Biomedical Research Involving Human Subjects [25] and Nuffield Council’s guidelines [26]. National and institutional research ethics policies and guidelines were built on the international principles and standards which were tailored to the local context. All guidelines for global research stipulated compliance with international and national laws and regulations and required ethics approvals in countries where research activities took place and in the country funding the study. Documents from HICs and LMICs highlighted the importance of respecting local societal norms, conducting research that benefits local communities and strengthens local capacities.

**Fig. 2:**
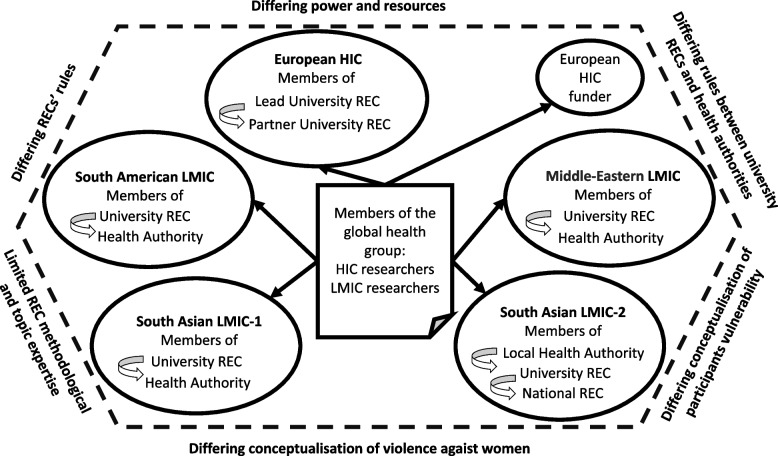
NER model for global research on health system responses to violence against women, research ethics committees’ approval stage

Our framework analysis generated 20 thematic codes, 12 candidate themes, and 3 final analytical themes summarising ethical challenges at the approval stage resulting from the global nature of the group, location of primary studies within health systems, and VAW topic. Within each theme, we reported perspectives on causes, impact, and solutions across the interviews and documents (Table 3, Fig. 2).

**Table 3:** Final analytical themes supported by interview and documentary data

Theme	Subtheme	Qualitative Interviews (*n* = 18)	Documents (*n* = 27)
1. Challenges resulting from the global nature of the group	1.1. Differing power and resources	11	Nuffield report 2002 [26] Helsinki Declaration 2013 [24] Nuffield Paper 2005 [27] Nuffield Report 2020 [28] CIOMS WHO Guidelines 2016 [25] [HIC funder] Policy.2021 [HIC funder] Policy.2021
	1.2. Differing RECs rules	13	Nuffield Paper 2005 [27] Nuffield Report 2020 [28] [HIC funder] Policy 2021 [HIC] University Guideline 2019
	1.3. Solutions to differing power, resources, and rules	12	CIOMS WHO Guidelines 2016 [25] Nuffield Paper 2005 [27] Nuffield Report 2020 [28] [LMIC] Government Legal 2013
2. Challenges resulting from the location of primary studies within health systems	2.1. Differing rules between university RECs and health authorities	4	[HIC funder] Policy 2021
	2.2. Solutions to differing rules between university RECs and health authorities	1	
3. Challenges resulting from the VAW topic	3.1. Differing conceptualisations of VAW	3	WHO Recommendations 2001 [29]
	3.2. Differing conceptualisations of vulnerability of research participants	4	WHO Recommendations 2001 [29] Helsinki Declaration 2013 [24] [LMIC] Government Legal 2013 [LMIC] Government Legal 2016 [HIC] University Guideline 2019 [HIC] University Policy 2019
	3.3. Limited REC methodological and topic expertise	5	
	3.4. Solutions to challenges resulting from the VAW topic	1	Nuffield Report 2002 [26]

*REC* Research Ethics Committee, *VAW* violence against women, [] anonymised country

### Challenges resulting from the global nature of the group

Location of research collaborators in HIC and several LMICs contributed to the challenges caused by factors in the following areas.

### Differing power and resources

Documentary and interview data suggested that hierarchical power imbalance between HIC and LMIC countries could be a contextual factor at the macro and meso levels. The power imbalance contributed towards ambiguity and frustration among researchers and REC members, and tensions between HIC and LMIC partners and between researchers, RECs and funders. The Nuffield report emphasised issues of power imbalance and the possible differences and conflicts between ethics committees in different countries [26]. In contrast, major European HIC funders of global research imposed ethics standards and processes that were based on their national laws as a global benchmark:

“[HIC funder name] is governed by [HIC name] law. The legislation supporting this policy relates to work carried out in the [HIC name]. We expect researchers to use similar standards and principles for any research outside the [HIC name]." ([HIC funder] Policy 2021).

Most interview participants perceived power imbalance between HICs (i.e., funder) and LMICs as a macro-level barrier. Some researchers and REC members felt that HIC policy makers and funders imposed their research agenda on LMICs, used funding as a mechanism for compliance, and set ethics regulations that did not suit the local context or were difficult to comply with because of limited research capacity and differing structures and resources in LMICs:

“It is much more likely when the project is managed by a general PI [principal investigator] of an institution from the global north, with funding from the global north, [that] countries from the global south that often participate with research participants and less with the thinking people, have much more difficulty to establish the limits and characteristics, the local peculiarities. So, I think it's more of a question of politics and power within research than the questions of Ethics Committees.” Interviewee 7, University REC and Hospital REC member, South American LMIC.

Some interview participants perceived the setting of funding priorities for global research by HICs without engaging with LMIC researchers and policy makers as a way of recolonising their research agenda. It was proposed that the solution was to engage local communities in research priority setting with funders:

“The only research funding is from international aid. Agencies become bureaucracies. In countries like our government doesn’t dictate policy, doesn’t set policy, it’s international aid that does. They dictate. Environment, this, that, that, what environment for god’s sake? We have so many wars here, every time we rebuild it gets destroyed. They set it in relation to their own priorities. It didn’t used to be like that. It used to be that agencies came, discussed, etc. and we together did things. Now, everything is on website, take it or leave it. The ethics is part and parcel of this approach of trying to say, “Wait a minute, do not colonise us that way too.” Interviewee 11, University REC member, Middle-Eastern LMIC.

While HIC funders mandated "that research performed in partner countries is conducted in accordance with regulations and to a standard no less stringent than those applicable in the [European HIC]" ([HIC funder] Policy 2021), some interviewees thought it was problematic due to the lack of consideration for the LMIC context in which research is being conducted. For example, LMIC and HIC researchers agreed that some LMIC universities did not have policies, processes, and resources for implementing stringent HIC requirements for data management. While their local RECs scrutinised the application sections about study design, methods, funding, they did not request the detailed data management plan which was an essential part of the HIC ethics applications.

### Differing RECs rules

The international and local policies and guidelines required ethics approvals from RECs in the funder country and in the countries where research activities took place. At meso level, the power was in the hands of multiple RECs in HIC and LMICs. Each REC interpreted and applied the universal ethics principles and international policies and guidelines differently. This resulted in the challenge of ‘differing RECs rules’ with varied requirements, processes, and timeframes which lacked coordination and challenged timely delivery of primary studies across LMICs. Researchers had to ‘problem-solve’ ethical approval conundrums themselves because RECs did not talk to each other and exercised power through standardisation of their approval process which many researchers described as highly bureaucratised, predominantly biomedical, and severely outdated. The power was in the RECs hands and researchers had to abide by the RECs rules with which they often did not agree. To mitigate these tensions, researchers used informal support from more experienced colleagues in their teams and partner countries, adapted study documentation previously approved by their RECs, and submitted ethics applications to the RECs “where you know people to make it easy” (Interviewee 13, University researcher, South-Asian LMIC). REC members followed their in-house standard operating procedures and best practice examples of previously approved research projects and felt that they adequately supported researchers throughout the application process. REC members thought that they treated local and global projects equally. They also thought that the international studies were more trustworthy because they had a higher level of funder scrutiny and the ability to recruit the best local experts.

Interviewees suggested measures at the macro level for mitigating power imbalances between HICs and LMICs. They proposed proactively lobbying HIC funders about LMIC priorities "to rethink research ethics in a way that is compatible with our local and regional [context]" (Interviewee 11, University REC member, Middle-Eastern LMIC). At the meso level, interviewees highlighted the importance of mutual learning and respecting country-specific contexts, transparent communication, and agreed partnership-wide practices for country-specific informed consent, safeguarding, data management, and helping each other with developing ethics applications and responding to RECs queries:

“Particularly in a global context, learning from others, because there is often a perception that it might be what people may consider the gold standard. It may not be, there may be more innovative ways to manage ethics and other regulatory approval processes from our global partners”. Interviewee 3, University REC member, European HIC.

To comply with the HIC funder’s requirements and improve their data safety, LMIC researchers wanted local policies for data management, additional funding to buy encrypted equipment and secure data storage, and education for local RECs and researchers on data management plans:

“…lobby and advocate for a strict data governance section within the [ethics application] pro forma that the ethical committee has." Interviewee 18, University researcher, South-Asian LMIC.

### Challenge resulted from the location of primary studies within health system

Location of primary studies in LMIC health care services was a contextual factor at the meso level which contributed to the challenge of reconciling the differing requirements and rules of both the university REC and health authority REC.

### Differing rules between university RECs and health authorities

Some LMIC researchers identified differing rules between university RECs and health authorities as a barrier which caused ambiguity and delays in local ethics approvals. To conduct research with health care professionals and patients, it was necessary to obtain ethics approvals from academic REC and ethics and/or regulatory approvals from the relevant health authority (e.g., Ministry of Health, Municipal health authority, healthcare setting). In two LMICs, this parallel process created an extra challenge for researchers. They experienced confusion, frustration, and delays because the two bodies had differing perspectives on the same issues and their approval processes were not coordinated:

“A problem that I always have, the university has a standard informed consent, and they understand that informed consent starts with a lot of data on the interviewee. When they send it to the municipality, the municipality says to me, "Oh, this is no good, this informed consent, we don't like it. You can't ask for all of this information," and I agree. Then I have to do something in between, because I have to negotiate with the two agencies, they ask for different things.” Interviewee 5, University researcher, South-American LMIC.

The conflicting rules could be explained by the varying perceived roles and responsibilities among research and health care approval bodies. Although REC members from universities and health authorities felt that they were responsible for the safety of research participants, the latter thought that they had better knowledge of their services and therefore an additional responsibility for the research participants as service users:

“Our concern is with protection of the users of our healthcare system within our jurisdiction, this is our chief concern. Even for very simple research the most important thing is how the municipality treat its healthcare service users. What guides us is mainly what the healthcare system means here in our city, because that is what we work with, the healthcare system users as research participants. So what guides us is the healthcare service, its logic, it’s dynamic, a research project can’t muddle with the services' dynamic or the work of the healthcare professionals.” Interviewee 6, Municipal REC member, South American LMIC.

Interviewees wanted more coordination between university and health system RECs which would harmonise and expedite the two approval processes.

" a coordination between the ethics board and the ministries itself. Like some kind of internal platform between the [cabinet work] and the policy level. I wish there was something like that so that the process would be a bit easier. Or some person from the ethics itself would be more cooperative and would help us to coordinate with them somehow so that the bureaucratic process is shortened." Interviewee 17, University researcher, South-Asian LMIC.

### Challenges resulted from the VAW topic

VAW as research topic was another factor at the macro and meso levels which required additional labour, time and resources for obtaining ethics approvals in LMICs. Documents and interview data identified additional VAW-specific challenges in the following areas.

### Differing conceptualisation of VAW

Sometimes the funder’s conceptualisation of VAW as a research topic differed from the local REC and researchers view due to the country-specific societal norms and political situation, resulting in ambiguity and frustration for researchers and REC members. One REC member from the LMIC experiencing protracted armed conflict thought that the conceptualisation of VAW in their country had been shaped primarily through the views of international aid agencies/research funders, with gender often being a substitute word for women and VAW being researched as an interpersonal problem in isolation from the chronic violence at the community and society levels. Such narrow conceptualisation could influence the choice of the research tools and produce biased findings. A researcher from the same LMIC explained that their country specific political and social context required researchers to defend their choice of international partners to get ethics approval for VAW research:

“VAW is perceived as a problem that should be treated on a local level. It is a sensitive topic rooted in the culture and religion. In our culture, religion, values, and traditions are strongly expressed and strongly engaged even within administration and research. You need to defend your research not only from an ethical point of view, but also from intention of why you’re doing this research with international partners.” Interviewee 10, University researcher, Middle-Eastern LMIC.

### Differing conceptualisation of vulnerability

While most documents and interviewees acknowledged that certain groups of research participants were more vulnerable than others and needed extra protection, only VAW-specific ethics guideline [29] and some experienced VAW researchers acknowledged vulnerabilities and protection for the researchers. According to REC members, they treated VAW like any sensitive topic and required proof of safeguarding and support resources for research participants. We found divergent views on the concept of vulnerability when applied in the VAW research context. In generic research ethics documents, vulnerability of research participants was defined as impairing their capacity to consent. Vulnerability meant that certain groups had limited ability to understand the nature of research and make informed decision about taking part, the possibility of being exploited and harmed by research. Several documents referred to vulnerable groups generically without providing specific definitions of clarifications regarding the types of individuals or conditions they encompassed. Other sources listed vulnerable groups, all of which were at risk of experiencing VAW – i.e., victims of traumatic events and sexual abuse, pregnant/lactating women, all women, women from orthodox communities, individuals disadvantaged by gender.

In contrast, VAW-specific ethics document [29], and some researchers from HIC and South Asian LMIC-2 recognised women who have experienced violence as capable of participating in research. The primary concern regarding women’s vulnerability in relation to participating in VAW research stemmed from the potential risk of experiencing further violence. Therefore, the protection measures ensured safety, confidentiality, and signposting to specialist VAW services. Researchers from two partner countries emphasised that all women who have experienced violence have agency and some of them are empowered by their lived experience. Therefore, that they should not be regarded as incapable of providing informed consent to participate in research. One researcher highlighted differing HIC and LMIC societal norms regarding vulnerability of research participants with the former fostering power among participants:

“I really like that the European context is stricter because it really ensures safety, security, privacy of the women. Especially when we are working with vulnerable groups. I think it comes out of respect for participants. Because I think in the [South Asian LMIC-2] context, vulnerable groups are sympathised and not empathised, maybe. Because out of sympathy you only feel pity for these women, and you don’t respect them as humans. When you respect a person then you would definitely think about how the researchers protect these women from being harmed or revictimized.” Interviewee 18, University researcher, South-Asian LMIC.

High-level guidelines and all interviewees acknowledged the need for additional time and effort for addressing issues of vulnerability in ethics applications through ensuring confidentiality, safety, and provision of referral/signposting to specialist VAW services. They highlighted the importance of the adequate time allocation for lengthy ethics approval processes which were sometimes delayed the commencement of the primary studies. Interviewees suggested allocating at least six months for obtaining ethics approvals.

"The special nature of this research topic [VAW] demands that safety concerns be considered from the very beginning of a study through its implementation and dissemination. This means that violence research will likely require a longer timeframe and a greater investment of resources to ensure these issues are fully addressed." (WHO Recommendations 2001 [29]).

From the perspective of healthcare REC, the established policies and care pathways should ensure the safety and appropriate care of patients affected by violence identified through studies on VAW and health:

“We have to read it [ethics application] carefully and make sure that if the researcher discovers that the woman is in fact experiencing violence it is notified in the national database. We have to be mindful of those things since they are health policies, the research project cannot go against our health policy, our care policy. We have a violence department here in the municipality, people that work solely with this, so a project like this has to know this exists and have a dialog with this area. We ask “what are you going to do when you see the person is experiencing violence? What care will you offer? How will you do it? What are you going to offer this person?”. We have to see if everything was thought of, otherwise this person will come here, do the research, get the data and just leave.” Interviewee 6, Municipal REC member, South American LMIC.

### Limited REC methodological and topic expertise

Researchers from the two South Asian LMICs felt frustrated with the limited VAW methodological and subject expertise of their RECs who dismissed qualitative and mixed methods, verbal informed consent, and remote data collection. They highlighted the importance of educating RECs and researchers on the specifics of the ethics applications for VAW research. For instance, one LMIC interviewee produced a resource sheet on VAW research for her institutional REC to support their ethics application and strengthen REC capacity. They noticed that their institutional REC application process improved over time with more VAW projects being undertaken. REC members also talked about continuing training and dialogue between RECs, RECs and researchers to strengthen capacity for ethical conduct of research. The interviewees agreed that the changes should occur at the institutional level:

“Simultaneously making sure ethics boards are having proper policies, guidelines, regulations, and qualified people who are able to review the ethics and provide substantial feedback to applicants. Not depending on who you know within the community and the ethics committee to push your application through.” Interviewee 14, University researcher, South-Asian LMIC.

## Discussion

This qualitative study of professional and policy perspectives generated three themes summarising and explaining challenges in global research on VAW and health at the ethics approval stage. The global nature of the research contributed towards differing power dynamics and resource distribution between HIC and LMICs and discrepant RECs rules across countries and institutions. HIC and LMIC researchers tried to mitigate the conflicting RECs rules by collaborating and supporting each other during the ethics application process. However, they lacked autonomy and capacity to shift the power from HIC or harmonise rules across RECs. Location of the primary studies in LMIC healthcare services contributed towards divergent institutional rules across academic RECs and health authorities that researchers tried to conciliate by negotiating the differences. The VAW topic contributed towards differing conceptualisations of VAW and participants vulnerability and limited methodological and topic expertise in some LMIC RECs which researchers addressed through helping REC to develop capacity.

These contextual factors had a negative impact on researchers and teams' morale, and the relationships between researchers, RECs, and HIC funders. Furthermore, they posed a substantial risk to the timely completion of studies. Most researchers felt frustrated and demotivated by the hierarchical, bureaucratised, uncoordinated, and lengthy approval processes. Participants suggested several strategies to address the power imbalances and challenges identified in the study. This included advocating for the involvement of LMIC representatives in shaping HIC funding agendas for global health research, prompting a redistribution of power between the HIC and LMICs at the macro- and meso- levels, fostering coordination between academic RECs and health authorities and between HIC and LMICs RECs, and prioritising capacity strengthening on ethics in VAW research. While these issues were present in all countries, their manifestations varied in terms of forms and degree due to the disparities in research infrastructure and healthcare systems.

Our analysis was informed by the NER model for global population health research [30] which we applied to the topic of global research on health system responses to VAW. Our study confirmed findings on ethical challenges in global health research reported in prior literature [14, 31] and discovered new challenges specific to the REC approval stage of studies on VAW as part of the global health agenda. These challenges were multifactorial and resulted from the global nature of the research group (disparities in power and resources, divergent RECs’ rules), location of primary study within LMIC health system (differing rules between university RECs and health authorities), and the topic of VAW (differing conceptualisation of VAW and vulnerability, limited methodological and topic expertise).

Our finding on the power asymmetry between HICs and LMICs as the major systemic driver of ethical challenges supports current discourse on decolonising research agendas and building equitable global health research partnerships [32]. While all interviewees and most high-level policies acknowledged power imbalance and advocated for equitable partnerships, researchers and REC members felt that HIC funders continued to dictate global health research agendas and impose their own institutional rules and societal norms on LMIC partners. Indeed, our interviewees perceived the agendas and rules prescribed by HIC funders and policy makers as a form of recolonisation which reinforced inequalities between HICs and LMICs at the macro level and jeopardised research integrity. Our global health research group tried to redress power imbalances through reflexivity about positionality during the research process which helped to establish and maintain equitable relationships within and between HIC and LMIC teams. However, our efforts at the meso (group) level could not change power asymmetry between HIC funder/RECs and LMIC researchers/RECs. As suggested by our findings and prior literature, rebalancing power requires interventions at the level of HIC policy makers and funders and HIC and LMIC RECs [10, 33].

Our finding on the challenge of disjointed academic RECs and health system authorities which imposed differing rules and lacked communication with each other is consistent with prior literature that found highly bureaucratised, disjointed, and lengthy ethics approval processes across HICs and LMICs [14]. Our interviewees’ suggestions for improving consistency and joined-up working amongst HIC/LMIC and academic RECs/health authorities support recommendations for more collaborative capacity strengthening and harmonisation across RECs in global research projects [34].

Our finding on differing conceptualisations of VAW reflects previous research that reported a lack of consensus regarding the definition of VAW and terminology used by researchers, practitioners, and research participants [35]. In the context of global research, the differences in definitions of VAW used by HIC funders, LMIC researchers and REC members were rooted in country-specific socio-political contexts. Some LMIC interviewees felt that HIC governments and funders imposed research agendas which defined VAW as a relationship problem and did not recognise the intersecting systemic violence and lived experience of people in a war torn LMIC. Nor do they acknowledge the ways in which political conflict can exacerbate different forms of gender-based violence. In contrast, LMIC participants living in countries affected by armed conflict acknowledged the complex interplay between individual, relationship, community, and societal factors that put people at risk of experiencing and using violence. This finding supports recommendations for inclusive agenda setting for global research, emphasising the importance of involving HIC funders, LMIC governments and researchers in setting priorities and co-designing research programmes that address the unique needs of LMICs and align with their socio-political contexts [33].

Our finding on the differing conceptualisations of vulnerability of research participants in global research on VAW could be explained by cultural variation regarding the concept of gender roles in different societies and the feminist ethos of VAW research. As highlighted by our interview participants, such conceptual differences have implications for the choice of research methodology and advocacy for participants. Feminist theories and approaches widely used and accepted in HICs might not offer a useful framework for transforming the realities of women experiencing violence in LMICs. Generalising their validity without contextual tailoring to LMIC-specific political and cultural contexts might hamper the very efforts to end violence [36]. Similarly, when applying methods and advocacy tools that have been developed in HICs to different LMICs, global research groups should consider the distinct context factors and actively seek the input of those who have local expertise and knowledge, to ensure the best recruitment, experiences of research participants and data generated [37].

The ethical debates surrounding the inclusion of women who experience violence as research participants revolve around ensuring their protection while also avoiding their undue exclusion from participating [38]. It is acknowledged that women who experience violence are not a homogeneous group, and therefore, considerations must be made to ensure their diverse experiences and perspectives are represented in research [39, 40].

Prior research has produced convincing evidence regarding the challenges in global research during ethics approval stage [34]. Future research should identify and evaluate policies and interventions that aim to address the causes of these challenges.

### Strengths and limitations

This study combined findings from qualitative interviews and complementary documentary analysis on ethics in global research on VAW and health. The use of qualitative methodology matched the objective of illuminating and contextualising the subjective experiences of researchers and REC members regarding obtaining ethics approval for global research projects. We added credibility to our findings by integrating results of interview and document analyses, involvement of three researchers from HIC and LMIC in data coding, whole team discussions of candidate and final analytical themes, and providing supporting quotes. We contributed towards transferability of our findings to similar contexts and participant groups through drawing a geographically diverse sample from one HIC and four LMICs across Europe, South America, Middle-Eastern region, and South Asia and through reporting socio-demographic characteristics of the participants. Table 1 shows that our purposive sampling strategy produced a maximum variation participant group in terms of countries, roles, years of relevant experience. However, the transferability has been limited by recruiting from the five partner countries within one global health research group. The sub-group from one South Asian LMIC did not include REC members.

Throughout the study, we critically examined and reflected on our own roles and influences of our values, assumptions, and experiences on the data we generated and analysis we produced. Our dual role as qualitative researchers and members of the global health group with direct experience of obtaining research ethics approvals allowed us to provided valuable insights, interpretations, and perspectives that contributed to the depth and richness of the findings. However, we acknowledge that the dual roles of author and research participant among three of the authors could also be seen as a limitation. It has the potential to introduce bias, as our perspectives may have influenced the interpretation of the findings. To mitigate this, we employed rigorous reflexivity practices, continuously interrogating our biases, and the impact of our involvement on the outcomes. Simultaneously, this insider perspective constituted a strength, enabling us to access and contribute to more nuanced understandings and ensure that researchers’ voices are accurately represented. In turn, this strengthened the credibility and relevance of the findings. It also helped to address potential prejudices and power imbalances because of the shared decision making in the interpretation and writing of the paper.

## Conclusions

Global research on health system responses to VAW generated additional challenges during application for ethics approvals across HIC and LMIC partners. These challenges were driven by power and resource asymmetries between HICs and LMICs, differing rules between RECs and between academic RECs and health authorities, varying conceptualisations of VAW and participant vulnerability, limited methodological and topic expertise in some LMIC RECs. The challenges had a negative impact on researchers’ relationships with RECs and funders. They imposed additional emotional labour on researchers and threatened timely delivery of the programme of the research. The process of ethics approval for global research on health system responses to VAW requires greater flexibility to accommodate country-specific contexts, with equal power distribution between HICs and LMICs, researchers and RECs. While some of these objectives can be achieved through educating individual RECs members, researchers, and funders, the power asymmetry and differing rules and contextualisation should be addressed at the meso (institutional) and macro (country) levels.

It is very important to conduct global research on health system responses to VAW to develop evidence-based interventions. Although a higher level of scrutiny during ethics approval stage might be justified, this should not hinder research on this topic, since findings are important to identify gaps in service provision and inform development of evidence-based interventions. By upholding high ethical standards in global research on health system responses to VAW, we ensure the opportunity for a comprehensive and evidence-based approach to addressing the issues. This, in turn, enhances the outcomes and results for women who have experienced violence.

### Supplementary Information

**Supplementary Material 1.**

## Data Availability

Due to the sensitivity of the data involved, these data are published as a controlled dataset at the University of Bristol Research Data Repository data.bris, at https://doi.org/10.5523/bris.3qs252vyomger219n9f5fcv5u3 [41]. The metadata record published openly by the repository at this location clearly states how data can be accessed by bona fide researchers. Requests for access will be considered by the University of Bristol Data Access Committee, who will assess the motives of potential data re-users before deciding to grant access to the data. No authentic request for access will be refused and re-users will not be charged for any part of this process.

## Abbreviations

HIC: High-income country
LMIC: Low- and middle-income country
REC: Research Ethics Committee
VAW: Violence against women
WHO: World Health Organisation
