# Supplementary material for: Ethical challenges in global research on health system responses to violence against women: a qualitative study of policy and professional perspectives
Source: BMC Med Ethics. 2024 Mar 19;25:32. doi: 10.1186/s12910-024-01034-y (PMC10949724; doi:10.1186/s12910-024-01034-y)
Supplement: Supplementary file 1 — Supplementary Material 1. [file 12910_2024_1034_MOESM1_ESM.docx]

# Interview topic guides

## Topic guide: researcher

1. Experience of preparing and obtaining ethics approval for [the global study on health system responses to violence against women].

- What was your role in preparing and obtaining ethics approval for [the global study on health systems responses to violence against women]
- Describe your local process for obtaining ethics approval for [the global study on violence against women] (for what research activities, which ethics committee(s)).
- Challenges (sensitive topic, global research, informed consent, adverse events, data management, differing policies across [global group on health system responses to violence against women] Universities).
- How these challenged were addressed (available resources: training on ethics in research – generic, violence against women, global research; help from colleagues).
- Policies/guidance on research ethics (high level, local) for global research, what they say, how they work in practice.
- What was it like to work with your university and other (e.g., Ministry of Health, hospital) research ethics committees (how did you feel)
- What motivated/facilitated your ethics work

1. Experience of obtaining ethics approval for other studies (local or global), on non-violence topic. If yes, compare with the [global study on health system responses to violence against women], how they differed, why.
2. Thoughts on how to negotiate differences in ethics processes across Universities and countries.
3. Impact of COVID on the process
4. How do you feel/what do you think about the purpose and the process for approval for a global research study.

## Topic guide: Member of research ethics committee

- Experience of reviewing ethics applications for global research, violence against women, [the global study of health system responses to violence against women].
- How are collaborative global research projects reviewed, approved, and monitored.
- Policies/guidance (high level, local), what they say, how they work in practice.
- Challenges (sensitive topic, global research, informed consent, adverse events, data management, differing policies across universities, countries).
- How these challenged are addressed (available resources: training on ethics in research – generic, violence against women, global research; help from colleagues)
- Experience of reviewing ethics applications for other studies global research on non-violence topics. How they differed, why.
- Thoughts on how to negotiate differences in ethics processes across universities and countries.
